# Effectiveness of the dementia nursing competence e-learning program for nurses in acute care hospitals in Japan

**DOI:** 10.1093/geroni/igab046.3011

**Published:** 2021-12-17

**Authors:** Mizue Suzuki, Tetsuro Ishihara, Nanayo Sasaki, Hiromi Yoshimura, Ikuko Sakai, Kimiyo Mastushita, Soichiro Mimuro, Keisuke Sawaki

**Affiliations:** 1 Hamamatsu Universuty school of medicne, Hamamatsu, Shizuoka, Japan; 2 Ishihara NeuroCognitive Clinic, seidai, Miyagi, Japan; 3 Hamamatsu University Hospital, Hamamastu, Shizuoka, Japan; 4 Institute for Graduate Nurses of Japanese Nursing Association, Tokyo, Tokyo, Japan; 5 Graduate School of Nursing Chiba University, chiba, Chiba, Japan; 6 Seirei Mikatahara General Hospital, Hamamastu, Shizuoka, Japan; 7 Hamamatsu University School of Medicine, Hamamastu, Shizuoka, Japan; 8 Hamamatsu Shugakusha Junior High School & High School, Hamamastu, Shizuoka, Japan

## Abstract

Objective: The number of older patients with dementia hospitalised in acute care hospitals increased and these patients underwent physical restrictions leading to a degeneration of essential mental and physical function. The dementia nursing competence e-learning program with audio-visual materials has been developed in acute care hospitals. Methods: An application form that explained the research was distributed to 1,944 registered nurses from seven hospitals, and 110 people applied. Nurses used an e-learning program for a month in May and practiced applying the knowledge learned from programs June through November 2020. The nurses completed a questionnaire survey at four periods: first (before program/baseline), second (after program), third (three months later), and fourth (six months later). In the second, third, and fourth periods, ‘Technical knowledge of the dementia nursing’ and ‘dementia nursing intervention’ were significantly improved as compared with the first. In the fourth period, ‘confidence of reduce of physical restriction’ showed significant improvement compared to the first. In ‘Ethical sensitivity scale of nurses’, the first of the four sub-scales significantly increased as compared with the first period. In ‘Self-assessment Scale of Nursing Practice for Elderly Patients with Cognitive Impairment with the Aim of Person-centred Care in Acute Care Hospitals’, the fourth sub-scale showed significant improvement compared to the first. In the ‘Personhood’ sub- scale of the Japanese version of Approaches to Dementia　Questionnaire, the fourth period showed a significant increase compared to the first. Conclusion: The results suggest that this program was effective and led to reduced physical restrictions in nursing practice.

